# Ruxolitinib Combined with Gemcitabine against Cholangiocarcinoma Growth via the JAK2/STAT1/3/ALDH1A3 Pathway

**DOI:** 10.3390/biomedicines9080885

**Published:** 2021-07-24

**Authors:** Shin-Yi Chung, Yi-Ping Hung, Yi-Ru Pan, Yu-Chan Chang, Chiao-En Wu, Dennis Shin-Shian Hsu, Peter Mu-Hsin Chang, Meng-Lun Lu, Chi-Ying F. Huang, Yeu Su, Michael Hsiao, Chun-Nan Yeh, Ming-Huang Chen

**Affiliations:** 1 Department of Oncology, Taipei Veterans General Hospital, Taipei 112201, Taiwan; u9910417@gmail.com (S.-Y.C.); yphong@vghtpe.gov.tw (Y.-P.H.); ptchang@vghtpe.gov.tw (P.M.-H.C.); violet306@gmail.com (M.-L.L.); 2 Institute of Clinical Medicine, National Yang Ming Chiao Tung University, Taipei 112304, Taiwan; 3 School of Medicine, National Yang Ming Chiao Tung University, Taipei 112304, Taiwan; 4 Department of Surgery, Chang Gung Memorial Hospital, Chang Gung University, Taoyuan 333423, Taiwan; panyiru0331@gmail.com; 5 Genomics Research Center, Academia Sinica, Taipei 115201, Taiwan; yuchanchang@ym.edu.tw (Y.-C.C.); mhsiao@gate.sinica.edu.tw (M.H.); 6 Department of Biomedical Imaging and Radiological Sciences, National Yang Ming Chiao Tung University, Taipei 112304, Taiwan; 7 Department of Hematology and Oncology, Chang Gung Memorial Hospital, Linkou and Chang Gung University, Taoyuan 333423, Taiwan; jiaoen@gmail.com; 8 Asclepiumm Taiwan Co., Ltd., New Taipei 25160, Taiwan; dennis660623@msn.com; 9 Institute of Biopharmaceutical Sciences, National Yang Ming Chiao Tung University, Taipei 112304, Taiwan; cyhuang5@ym.edu.tw (C.-Y.F.H.); yeusu@ym.edu.tw (Y.S.)

**Keywords:** cholangiocarcinoma, ALDH1A3, JAK2 inhibitor, STATs, ruxolitinib

## Abstract

Cholangiocarcinoma is the most common primary malignant tumor of the bile duct. The current standard first-line treatment for advanced or metastatic cholangiocarcinoma is gemcitabine and cisplatin. However, few effective treatment choices exist for refractory cholangiocarcinoma, and additional therapeutic drugs are urgently required. Our previous work demonstrated that the ALDH isoform 1A3 plays a vital role in the malignant behavior of cholangiocarcinoma and may serve as a new therapeutic target. In this study, we found a positive correlation between ALDH1A3 protein expression levels and the cell migration abilities of three cholangiocarcinoma cell lines, which was verified using ALDH1A3-overexpressing and ALDH1A3-knockdown clones. We also used ALDH1A3-high and ALDH1A3-low populations of cholangiocarcinoma cell lines from the library of integrated network-based cellular signatures (LINCS) program and assessed the effects of ruxolitinib, a commercially available JAK2 inhibitor. Ruxolitinib had a higher cytotoxic effect when combined with gemcitabine. Furthermore, the nuclear translocation STAT1 and STAT3 heterodimers were markedly diminished by ruxolitinib treatment, possibly resulting in decreased ALDH1A3 activation. Notably, ruxolitinib alone or combined with gemcitabine led to significantly reduced tumor size and weight. Collectively, our studies suggest that ruxolitinib might suppress the ALDH1A3 activation through the JAK2/STAT1/3 pathway in cholangiocarcinoma, and trials should be undertaken to evaluate its efficacy in clinical therapy.

## 1. Introduction

Intrahepatic cholangiocarcinoma is a relatively rare hepatobiliary cancer that is becoming increasingly common [1,2,3]; it is an aggressive cancer, typically diagnosed at an advanced stage and has a poor prognosis [4]. It originates from the bile duct epithelium and can be found anywhere along the biliary tree. Cholangiocarcinoma is classified as intrahepatic or extrahepatic, and most cases are adenocarcinoma. It is rare compared with hepatocellular carcinoma. Several molecular targeted therapies have been assessed, with the median progression-free survival being relatively low at 1.8–7 months [5,6]. However, the incidence and mortality have increased in recent years, especially with intrahepatic cholangiocarcinoma, at least partly due to the improvement of diagnostic tools, such as imaging modalities, molecular diagnosis, and pathology techniques. In other words, the increase may only reflect the numbers that were previously misclassified as malignancy of unknown origin or hepatocellular carcinoma.

Complete resection is currently the only potentially curative treatment for patients with resectable disease; however, most patients are not a candidate for surgery because they are often diagnosed when the tumor is at an advanced stage. Furthermore, tumor recurrence is common after resection, even in patients with a negative margin [7,8,9]. Therefore, chemotherapy is an option for these unresectable or relapse/recurrence patients. Several phase II and phase III studies have demonstrated a survival benefit with a gemcitabine-based regimen [10,11,12,13]. A randomized, controlled phase III trial enrolling 410 patients with locally advanced or metastatic biliary tract cancer demonstrated that gemcitabine and cisplatin improved overall survival and progression-free survival by 30% compared with Gemcitabine alone [13]. The overall survival in the combination group was 11.7 months. Several candidate oncogenic alterations have been identified with the potential for drug-based treatments [14], and pemigatinib, which targets fibroblast growth factor receptor 2 (FGFR2) rearrangement or fusion, has been developed. A multicenter, open-labeled, single-arm trial found pemigatinib to be effective in patients with advanced cholangiocarcinoma, and the FDA approved pemigatinib for cholangiocarcinoma [15].

Recent evidence suggests that enhanced aldehyde dehydrogenase (ALDH) activity is associated with increased drug resistance and metastasis in various cancers. Our previous work demonstrated that the ALDH superfamily, especially 1A3, plays a vital role in malignant behavior and gemcitabine resistance in patients with advanced cholangiocarcinoma [16]. In the current study, we used an L1000 system and computational approach to determine potential agents for cholangiocarcinoma treatment [16]. We also verified the efficacy of these agents using cell lines and animal models.

## 2. Material and Methods

### 2.1. Cell Culture

A human immortalized cholangiocyte cell line, MMNK1, and several cholangiocarcinoma cell lines, namely KKU-M055, KKU-100, KKU-M213, and HuCCT1, were obtained from the Japanese Collection of Research Bioresources Cell Bank (JCRB; Osaka, Japan). Cells were cultured in Dulbecco’s modified Eagle’s medium (DMEM; Gibco; Thermo Fisher Scientific, Waltham, MA, USA). All mediums were supplemented with 10% fetal bovine serum (GIBCO, Grand Island, NY, USA), penicillin (100 units/mL), and streptomycin (100 μg/mL). Cells were incubated in a humidified atmosphere at 37 °C with 95% air and 5% CO_2_.

### 2.2. Virus Production and Infection

shALDH1A3 clones were amplified from the pGIPZ gene bank (Thermo company, from Dr. Michael Hsiao’s library). Plasmids were transfected into 293T cells with pCMV△8.91 and pMD.G. Target cells were seeded at an appropriate density in a 10 cm dish 24 h before infection. On the second day of infection, the growth medium was changed, and virus supernatants were collected after 48 and 72 h. A combination of a virus supernatant and polybrene (final concentration: 8 µg/mL) was used for various cell models. Subsequently, the plate was incubated for 24 h. After incubation, the medium was removed, and a fresh medium containing puromycin was added. Approximately 72 h after infection, cells were further split, and the selection was continued until all control cells were dead.

### 2.3. Transwell Migration Assay

Cholangiocarcinoma cells were trypsinized and resuspended in a serum-free DMEM medium and adjusted to a density of 1.5 × 10^5^ cells/mL. In the bottom chamber, 600 μL of complete medium was placed, and 200 μL of the cell suspensions described earlier were seeded in the top chamber. The cells were allowed to migrate for 16 h and were then fixed with 1% formaldehyde, followed by staining with 0.005% crystal violet. Before light microscope examination (Olympus IX70, 10× objective), the cells remaining in the top chamber were removed using cotton swabs. Three random fields were photographed for each Transwell. The average number of migrated cells per field was calculated using MetaMorph software (Universal Imaging Corporation, New York, NY, USA) based on the results of three independent experiments.

### 2.4. L1000 and LINCS Analysis

L1000 is an innovative gene expression profiling technique with a high-throughput scale (20 × 384 samples per week) for next-generation pharmaceutical discovery applications. It includes 3000 human genes, known targets of FDA-approved drugs, drug–target pathway components, and candidate disease genes. Using L1000 profiling, disease indications can be linked with potential lead compounds, and genetic perturbagens can be generated using dedicated pattern-matching algorithms in the Library of Integrated Network-based Cellular Signatures (LINCS; http://www.broadinstitute.org/LINCS/dataset.html, accessed on 18 June 2021). Table S1 lists the gene perturbagen candidates, which were generated from a query of the expression level of ALDH-low and ALDH-high genes in the HuCCT1 cell signature in the LINCS program.

### 2.5. Cell Viability Measurements

Cell viability was determined using an MTT cell viability assay kit (Trevigen, Gaithersburg, MD, USA), according to manufacturer’s protocols. The cells were seeded at 2000 cells/100 µL culture medium/well in 96-well microplates. At 24 h after seeding, the cells were treated with various concentrations of gemcitabine dissolved in dimethyl sulfoxide with or without 10 μm of ruxolitinib for 72 h in a humidified atmosphere containing 5% CO_2_ at 37 °C. Subsequently, the cells were incubated in a medium containing MTT for 4 h and were lysed with dimethyl sulfoxide; the optical density of the resulting supernatant was measured at 570 nm by using a microplate reader (Spectral Max 250; Molecular Devices, Sunnyvale, CA, USA).

### 2.6. Western Blot Analysis

Cells were lysed at 4 °C in RIPA buffer supplemented with protease and phosphatase inhibitors. Equal loads of 30 μg of proteins were electrophoretically separated using SDS/polyacrylamide gels and then transferred to the PVDF membrane (Millipore, Bedford, MA, USA). After blocking with 5% nonfat milk, the membrane was made to react with specific antibodies (ALDH1A3, Abcam #ab129825, 1:1000; STAT3, Cell Signaling #4904, 1:1000; p-STAT3 (Try705), Cell Signaling #9145, 1:1000; STAT1, Santa Cruz #sc-417, 1:1000; p-STAT1 (Try701), Cell Signaling #9167, 1:1000; β-actin, Sigma-Aldrich #A2228, 1:1000) overnight at 4 °C and then incubated with horseradish peroxidase-conjugated secondary antibody for 1 h. Blots were visualized using an ECL-Plus detection kit (PerkinElmer Life Sciences, Boston, MA, USA).

### 2.7. Immunohistochemistry Staining

Cholangiocarcinoma specimens from patients were fixed in formalin, embedded in paraffin, sliced into 4 μm thick sections, and then stained for selected markers. Primary antibodies (ALDH1A3, 1:500, GeneTex GTX110784; p-STAT1, 1:100, Cell Signaling #9167; p-STAT3, 1:200, Cell Signaling #9145) were incubated overnight at 4 °C. The control slides were incubated in diluent without the primary antibody. The slides were then washed three times for 5 min each in TBST prior to visualization using the REAL EnVision Detection System, Peroxidase/DAB+, Rb/Mo (K500711, DAKO). After 3 TBST washes for 5 min each, the slides were counterstained with hematoxylin, mounted, and then blindly analyzed using a microscope.

### 2.8. Quantitative RT-PCR

Total RNA was isolated from cells using TRIzol reagent (MDBio, Taipei, Taiwan), and 5 μg of the RNA was reverse-transcribed using MMLV RT (Thermo Fisher Scientific Waltham, MA, USA). SYBR Green-based quantitative PCR was then performed using the CFX Connect™ Real-Time PCR Detection System (Bio-Rad, Hercules, CA, USA) with primer sets designed to analyze the expression of specific genes, including *RELA:* forward: 5′-GCGAGAGGAGCACAGATACC-3′ and reverse: 5′-CTGATAGCCTGCTCCAGGTC-3′; *HIF1A:* forward: 5′-GAAAGCGCAAGTCCTCAAAG-3′ and reverse: 5′-TGGGTAGGAGATGGAGATGC-3′; *SRF:* forward: 5′-GCCACTGGCTTTGAAGAGAC-3′ and reverse: 5′-GGTGCCAGGTAGTTGGTGAT-3′; *FOXP3:* forward: 5′-CATGATCAGCCTCACACCAC-3′ and reverse: 5′-CCACTTGCAGACACCATTTG-3′; *ATF1:* forward: 5′-CAACCTGGTTCAGCAGTTCA-3′ and reverse: 5′-TTTCTGCCCCGTGTATCTTC-3′; *SP1:* forward: 5′-TCATACCAGGTGCAAACCAA-3′ and reverse: 5′-GCTGGGAGTCAAGGTAGCTG-3′; *STAT1:* forward: 5′-CCGTTTTCATGACCTCCTGT-3′ and reverse: 5′-TGAATATTCCCCGACTGAGC-3′; *IRF1:* forward: 5′-AGCTCAGCTGTGCGAGTGTA-3′ and reverse: 5′-TAGCTGCTGTGGTCATCAGG-3′; *JUN:* forward: 5′-CCCCAAGATCCTGAAACAGA-3′ and reverse: 5′-CCGTTGCTGGACTGGATTAT-3′. The reaction conditions were as follows: 95 °C for 10 min, 40 cycles at 95 °C for 30 s and 65 °C for 30 s, and 72 °C for 30 s. The relative target gene expression was calculated using the comparative Ct method (ΔΔCt), which was normalized to endogenous GAPDH levels using CFX Manager version 3.1 (Bio-Rad).

### 2.9. Re-Chromatin Immunoprecipitation Assay

Re-chromatin immunoprecipitation (ChIP) assays were performed following the protocol provided by SimpleChIP Enzymatic Chromatin IP Kit (Magnetic Beads) (Cell Signaling #9003). Briefly, KKU-100 and KKU-M213 cells were treated with or without 10 μm of ruxolitinib for 7 and 3 h, respectively. Cells were fixed with 1% formaldehyde for 10 min, followed by 5 min treatment with 10× glycine to quench the reaction. Nuclear extracts were prepared with buffers A and B, digested using micrococcal nuclease, and then sonicated to shear DNA into 150–900-bp fragments. Precleared lysates (3 μg) were subjected to overnight immunoprecipitation with 2 μg/mL histone H3 (D2B12) XP Rabbit mAb (#4620) (positive control), normal rabbit IgG (#2729), or rabbit p-STAT1 (Try701) (Cell Signaling #9167). Next, the lysates were incubated with protein G magnetic beads. The eluted chromatin was used for the re-ChIP assays and was added to antibody bead complexes containing 2 μg/mL p-STAT3 (Try705) (Cell Signaling #9145) for 24 h under rotation at 4 °C. DNA samples were purified using a DNA purification spin column. A volume equal to that of the final precipitate was used for PCR amplification with primers (primer 1: forward: 5′-GGAGCCCCGGATTTGCAGAAGCAG-3′ and reverse: 5′-ATGGCCTGGAGTGCACAGCTGAGG-3′; primer 2: forward: 5′-GCACTCCAGGCCATAACAGCACAA-3′ and reverse: 5′-GTAATCAACAATGGGGAGGGGTCT-3′; primer 3: forward: 5′-AAGCCGCTTCCGTGTCTCAGATGG-3′ and reverse: 5′-TTACTTCCCAGGAGGGGGAGGTAA-3′) and an ALDH1A3 promoter region amplicon containing a putative STAT1-binding site.

### 2.10. Animal Experiments

Animal studies were performed with the approval of the Academia Sinica Institutional Animal Care and Utilization Committee (IACUC) or the Institutional Animal Care and Use Committee (IACUC) (IACUC No. 12-02-319)of Chang Gung Memorial Hospital. In addition, all animal studies followed the US National Institutes of Health Guidelines for the Care and Use of Laboratory Animal protocols (Publication No. 85–23, revised 1996). Age-matched severe combined immunodeficiency gamma (JAX™ NOD.Cg-Prkdcscid Il2rgtm1Wjl/SzJ; NOD-SCIDγ) male mice aged 6 weeks were used (Jackson Laboratory, Bar Harbor, ME, USA). A total of 5 × 10^6^ SNU-308 cholangiocarcinoma cells were resuspended in 100 μL of PBS and injected subcutaneously under the dorsal skin of the mice. The administration and concentration were modified from previous studies [17,18]. Mice were divided into groups and randomized to different treatments as follows: control group; gemcitabine (50 mg/kg, twice per week, once every 2 weeks (Q2W), intraperitoneal injection); ruxolitinib (60 mg/kg, twice per day, orally); and gemcitabine + ruxolitinib. Ruxolitinib monophosphate was formulated in 0.5% HPMC (Pharmacoat 603, Dow Chemical) at a concentration of 7.9 mg/mL and was administered orally twice per day at 12 h intervals at 10 mL/kg as a free-base equivalent dose of 60 mg/kg. The tumor volumes and body weights of mice were measured once weekly, and tumor masses were harvested after 5 weeks. Tumor growth was monitored once weekly by using Vernier caliper measurement of two perpendicular tumor diameters (L and W). Tumor volume was calculated using the formula LW^2^/2.

### 2.11. Promo 3.0

The putative ALDH1A3 promoter region from −1500 to +100 relative to transcription start site was analyzed using the PROMO 3.0 software (http://alggen.lsi.upc.es/cgi-bin/promo_v3/promo/promoinit.cgi?dirDB=TF_8.3, accessed on 18 June 2021) to predict the six possible transcription factor binding sites. The maximum matrix dissimilarity rate was 1.

### 2.12. Positron Emission Tomography

Position emission tomography (PET) was performed as described previously [19] at the Center for Advanced Molecular Imaging and Translation, Chang Gung Memorial Hospital, Linkou (Taoyuan, Taiwan). In brief, rats were treated with TAA, before being subjected to serial PET scanning at 2 and 4 weeks using the Inveon™ system (Siemens AG, Munich, Germany). Animals were assigned to the control and treatment groups based on their baseline PET results to ensure similar PET-positive rates in the two groups. The details of radioligand preparation, scanning protocols, and determination of optimal scanning time have been previously described by our group. In brief, animals were fasted overnight prior to scanning, and 90 min after 18F-FDG intravenous injection, 30 min static scans were obtained for all the animals. All imaging studies on animals were performed at 37 °C under anesthesia (2% isoflurane vaporized in 100% oxygen) on a controlled imaging bed (Minerve System, Esternay, France). PET images were reconstructed using the two-dimensional ordered subset expectation-maximization method (4 iterations and 16 subsets) without attenuation or scatter corrections. All imaging data were processed by the PMOD image analysis workstation (PMOD Technologies, Zurich, Switzerland).

The largest liver tumor for each animal was identified through careful investigation of the three tumor image sets obtained for each rat. The uptake of 18F-FDG by the normal and tumor liver tissues was quantified by calculating the standardized uptake value (SUV). The SUVs were calculated according to the recommendations of the European Organization for Research and Treatment of Cancer. Regions of interest (ROIs) in the tumors were determined by obtaining transverse images of the selected tumors and measuring the largest diameter. The normal liver ROIs were also determined by measuring the diameter of transverse images obtained from the normal liver tissues. The mean SUV (SUVmean) of the normal and tumor liver tissues was calculated, and the respective tumor-to-liver (T/L) radioactivity ratios were compared for the two tissue types.

### 2.13. Statistical Analysis

All data are presented as the mean ± standard deviation. Differences between the experimental and control groups were calculated using the Student’s *t* test. Progression-free survival and overall survival rates were evaluated using the Kaplan–Meier method. Several clinicopathologic variables were considered for the initial univariate analysis, which was performed using the log-rank test. The Cox proportional hazards model was applied for multivariate regression. SPSS for Windows (Version 17.0, Chicago) was used for statistical analysis. A two-tailed *p* value of ≤0.05 was considered statistically significant.

## 3. Results

### 3.1. ALDH1A3 Affects the Migration Abilities of Cholangiocarcinoma Cells

**Figure 1:**
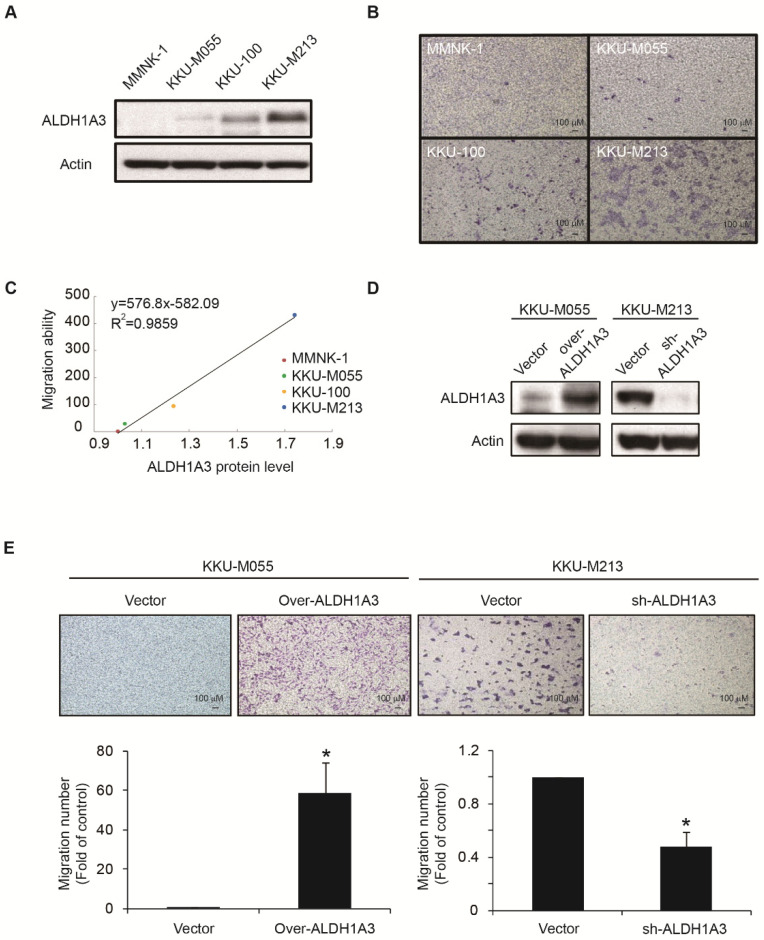
ALDH1A3 expression influences the migration ability in cholangiocarcinoma cell lines. (**A**) Total lysates (20 μg) from the normal cholangiocyte cell line (MMNK-1) and three cholangiocarcinoma cell lines (KKU-M055, KKU-100, and KKU-M213) were subjected to Western blot analysis using an anti-ALDH1A3 antibody as the probe, and β-actin signals were used as loading controls. (**B**) The cell migration ability of all cell lines was analyzed using Transwell migration assays. (**C**) Correlation of protein expression levels of ALDH1A3 with migration abilities. (**D**) Total lysates (20 μg) prepared from the vector- and ALDH1A3-overexpressing KKU-M055 cells and vector- and ALDH1A3-knockdown KKU-M213 cells, respectively, were subjected to Western blot analyses using antibodies against ALDH1A3. β-actin signals were used as loading controls. (**E**) The cell migration abilities of all KKU-M055 and KKU-M213 cells were analyzed using Transwell migration assays. The quantitative results shown in the bottom panels are the mean ± standard deviation of three independent experiments. * *p* < 0.05 compared with the respective vector cells by the Student’s *t* test.

To investigate the endogenous levels of ALDH1A3 in normal cells and tumor cells, we evaluated the protein expression level of ALDH1A3 in MMNK1, which is an immortalized human normal bile duct cell line, and in several cholangiocarcinoma cell lines—KKU-M055, KKU-100, and KKU-M213 (Figure 1A). The cholangiocarcinoma cell lines expressed higher ALDH1A3 than the normal MMNK-1 cells. The migration assay demonstrated that the migration ability (Figure 1B) was positively correlated with the level of ALDH1A3 (Figure 1C). To further examine ALDH1A3 expression and cell migration abilities, we established an ALDH1A3-overexpressing KKU-M005 cell line and knocked down ALDH1A3 in the KKU-M213 cell line, which expressed the highest ALDH1A3 levels among the three cholangiocarcinoma cell lines (Figure 1D). As expected, the migration abilities of the ALDH1A3-overexpressing KKU-M055 cells was >50 times higher than that of the vector control. By contrast, ALDH1A3 knockdown decreased cell migration by approximately 50% (Figure 1E). These results suggested that ALDH1A3 positively affects cholangiocarcinoma cell migration.

### 3.2. Identify Potential Drugs to Reverse the Gene Signatures of ALDH1A3 in Cholangiocarcinoma Cell Lines

**Figure 2:**
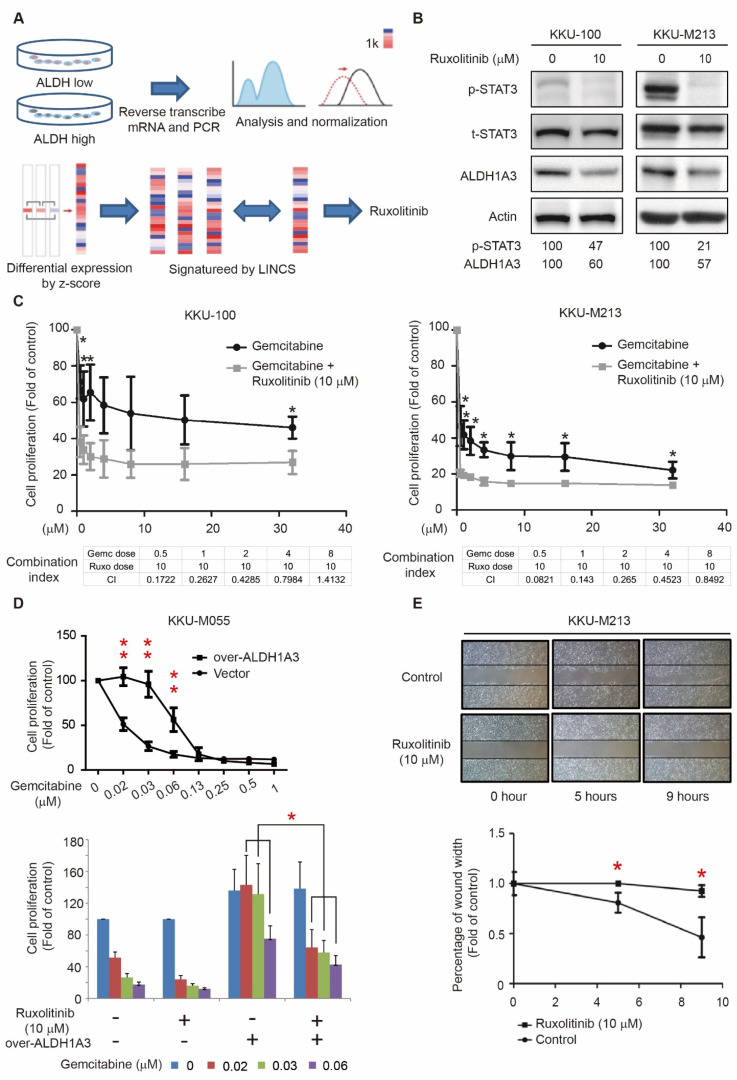
Ruxolitinib had a synergistic effect with gemcitabine and inhibited cholangiocarcinoma cell migration. (**A**) The inhibitors were identified as potential drugs. (**B**) Total lysates (20 μg) from the KKU-100 and KKU-M213 cells were subjected to Western blotting analyses using primary antibodies against ALDH1A3, STAT3, and p-STAT3 as probes. β-actin signals were used as loading controls. (**C**) The KKU-100 (left, upper) and KKU-M213 (right, upper) cells were seeded onto 96-well plates and treated with gemcitabine alone or various concentrations of gemcitabine plus 10 μM ruxolitinib for overnight incubation. At 48 h after treatment, cell viability was measured using an MTT assay and was normalized to the respective untreated cells. The combination index (CI) values of the interaction between gemcitabine plus ruxolitinib against KKU-100 (left, bottom) and KKU-M213 (right, bottom). Data shown are the averages of three independent experiments. * *p* < 0.05 compared with the untreated cells with the same treatments by Student’s *t* test. (**D**) The vector- and ALDH1A3-overexpressing KKU-M055 cells were seeded onto 96-well plates and treated with gemcitabine alone (upper) or various concentrations of gemcitabine plus 10 μM ruxolitinib (bottom) for overnight incubation. At 48 h after treatment, cell viability was measured using an MTT assay and was normalized to the respective untreated cells. Data shown are the averages of three independent experiments. * *p* < 0.05, ** *p* < 0.01 compared with the vector cells with the same treatments by Student’s *t* test. (**E**) Cell migration abilities were analyzed using a wound-healing assay. Representative images reveal KKU-M213 cells treated with 10 μM ruxolitinib compared with the control after 0, 5, and 9 h. The quantitative results shown in the bottom panels are the mean ± standard deviation of three independent experiments. * *p* < 0.05 compared with the respective vector cells by the Student’s *t* test.

In a previous study, we established the ALDH1A3-overexpressing and -knockdown HuCCT1 clones as the ALDH^high^ and ALDH^low^ cells, respectively, and determined how ALDH expression affects their migration abilities [16]. To identify drugs that can inhibit cell migration, we utilized the LINCS program to analyze the gene signatures of ALDH^high^ and ALDH^low^ HuCCT1 cells and obtained the potential drug, ruxolitinib, a JAK2 inhibitor (Figure 2A, Table S1). To validate the LINCS results, we treated KKU-100 and KKU-M213 cells with 10 μM ruxolitinib for 8 and 3 h, respectively; ruxolitinib inhibited the expression of ALDH1A3 (Figure 2B). In addition, we examined the expression level of STAT3 and its phosphorylation and found ruxolitinib to effectively inhibit STAT3 activation.

We then investigated whether ruxolitinib has a synergistic effect with the existing clinical drugs, such as gemcitabine, for the treatment of cholangiocarcinoma. The cell proliferation assay demonstrated that the combination of gemcitabine and ruxolitinib significantly decreased cell viability in both KKU-100 and KKU-M213 cell lines (Figure 2C and Figure S1). Furthermore, we examined the anti-tumor effect on the vector and ALDH1A3-overexpressing KKU-M055 cells; the overexpression of ALDH1A3 in KKU-M055 cells increased the gemcitabine resistance. Nevertheless, the result of ruxolitinib and gemcitabine combination treatment significantly reduced cell proliferation compared to gemcitabine alone in ALDH1A3-overexpressing KKU-M055 cells (Figure 2D). Additionally, the migration ability increases in the ALDH1A3-overexpressing KKU-M055 cells and could be reduced when treated with ruxolitinib, suggesting ruxolitinib inhibits cell migration via suppressing ALDH1A3. Due to the lower endogenous level of ALDH1A3 in KKU-M055 cells, the migration ability is not significantly different with treated ruxolitinib (Figure S2). Collectively, these results showed that the anti-tumor effect of ruxolitinib on cholangiocarcinoma partially depends on ALDH1A3.

Because ALDH1A3 enhances the cell migration ability in cholangiocarcinoma cell lines (Figure 1), we performed the wound-healing assay to examine whether ruxolitinib, an ALDH1A3 inhibitor, inhibited migration. We found that ruxolitinib strongly suppressed cell migration after treatment for 5 and 9 h (Figure 2E). We also used an STAT3 inhibitor, cryptotanshinone, to treat cholangiocarcinoma cell lines. Cryptotanshinone inhibited ALDH1A3 expression and cell migration, as well as inhibiting cholangiocarcinoma cell growth synergistically with gemcitabine (Figure S3). Taken together, these findings imply that ruxolitinib can inhibit cholangiocarcinoma cell migration and has a synergistic antitumor effect with gemcitabine.

### 3.3. Expression of ALDH1A3 through Nuclear Translocation of STAT1 and STAT3 Heterodimer

**Figure 3:**
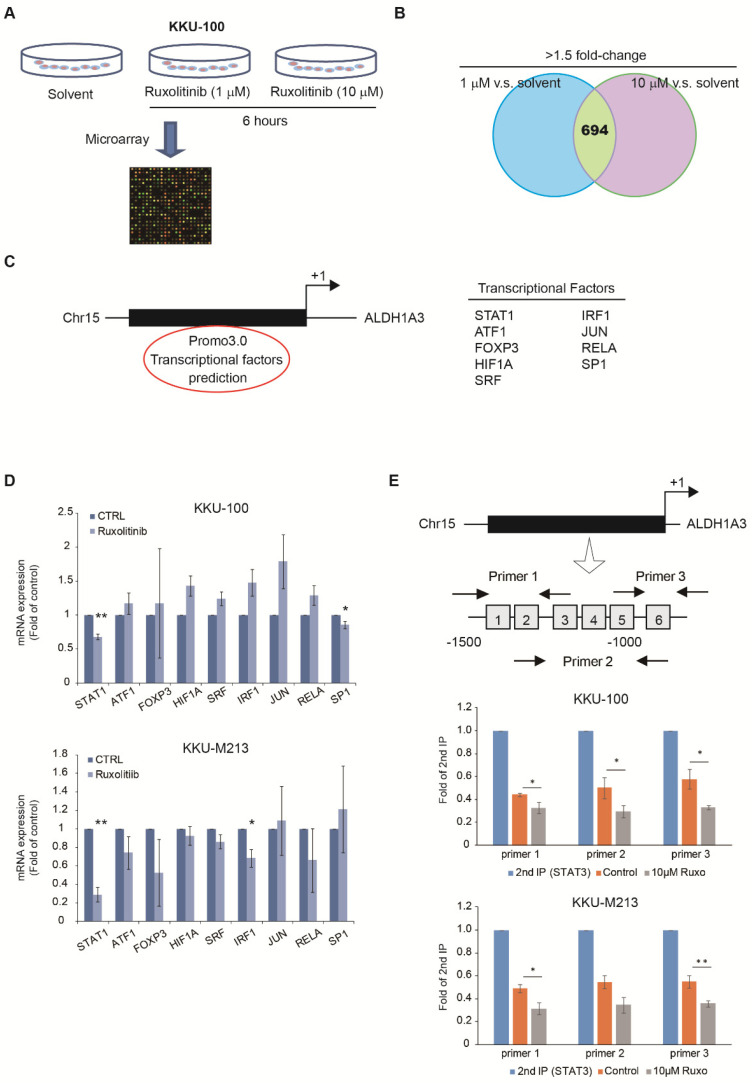
Effects of ALDH1A3 via functions of STAT1 and STAT3. (**A**) The gene signatures of KKU-100 cells cultured for 6 h with or without low doses (1 μM) or high doses (10 μM) of ruxolitinib were analyzed using Ingenuity Pathway Analysis (IPA). (**B**) The scheme of different gene signatures of KKU-100 cells was validated by IPA. (**C**) The nine putative transcription factors that correlated with ALDH1A3 and ruxolitinib were predicted using the PROMO 3.0 software. The lists of transcription factors are listed in the right panels. (**D**) Total RNA (5 μg) was isolated from the KKU-100 and KKU-M213 cells and subjected to RT–qPCR analyses to determine the mRNA levels of predicted transcription factors, including *STAT1*, *ATF1*, *FOXP3*, *HIF1A*, *SRF*, *IRF-1*, *JUN*, *RELA*, and *SP1*. Data are the mean ± standard deviation of three independent experiments. * *p* < 0.05 and ** *p* < 0.01 compared with the respective control group by the Student’s *t* test. (**E**) Schematic of the upstream promoter region of the ALDH1A3 gene with six predicted STAT1-binding sites (1–6). The region amplified in the re-ChIP assay is indicated by arrows (primers 1–3). The DNA samples represent yields of the secondary immunoprecipitation (second IP), control, and drug-treated groups (anti-pSTAT1 and p-STAT3). Data are the mean ± standard deviation of three independent experiments. * *p*  <  0.05 and ** *p*  <  0.01 compared with the respective control group by the Student’s *t* test.

We used in silico methods to explore the correlation between the JAK2/STAT3 signaling pathway and ALDH1A3. Total RNA was extracted from ruxolitinib-treated cells and sham groups of KKU-100 cells. The raw intensities were normalized, and a fold change of >1.5 times in the ruxolitinib group (high/low dose) compared with solvent control (694 probes) was set as the cutoff to predict potential upstream regulators by using GeneSpring software (Agilent Technologies) (Figure 3A). We explored common signatures between high-dose (10 μM) ruxolitinib versus the control with low-dose (1 μM) ruxolitinib versus control in a cell model, and we calculated the Z score and P value of several candidate targets by using the Ingenuity Pathway Analysis tool (Figure 3B, Table S2). The combination of these methods narrowed the potential transcriptional factors of ALDH1A3. On the other hand, we noticed that ALDH1A3 is the one of the significant change downstream factors in a dose-dependent manner in our profiles. Therefore, we analyzed potential transcription factors and may directly bind to the promoter region of ALDH1A3. The potential transcription factor binding sites on the ALDH1A3 promoter region (–1500 to +1 base pairs relative to the transcription start site) were searched using the PROMO 3.0 software, and nine such sites were identified: *STAT1*, *ATF1*, *FOXP3*, *HIF1A*, *SRF*, *IRF1*, *JUN*, *RELA*, and *SP1*. (Figure 3C). To investigate whether these potential transcription factors respond to ruxolitinib, we analyzed their mRNA levels using RT–qPCR after treatment with 10 μM ruxolitinib and found a decrease in the mRNA level of only STAT1 in both KKU-100 and KKU-M213 cells (Figure 3D). SP-1 and IRF-1 decreased in KKU-100 and KKU-M213 cells, respectively.

Because previous studies have demonstrated that the expression of STAT1 and STAT3, which form the STAT1/3 DNA complex heterodimer, is a critical marker for the diagnosis and prognosis in colorectal cancer (CRC) [20], we evaluated whether STAT1/STAT3 play a similar role in regulating the expression of ALDH1A3 in cholangiocarcinoma. No STAT3 binding site exists on the ALDH1A3 promoter region; therefore, we searched the six putative STAT1 binding sites on the ALDH1A3 promoter and designed three different primers to involve one to three binding sites. The results of the re-ChIP assay indicated that ruxolitinib diminished the binding of the STAT1/3 complex on the ALDH1A3 promoter region (Figure 3E). Interestingly, primer 3, which involved putative binding site 6, had the strongest response, suggesting that it is the essential binding site of the STAT1/3 DNA complex heterodimer. Our findings imply that ruxolitinib inhibits the ALDH1A3 expression via the JAK2/STA1/3 pathway.

### 3.4. Combination of Ruxolitinib and Gemcitabine Conduces to Cholangiocarcinoma Tumor Shrinkage

**Figure 4:**
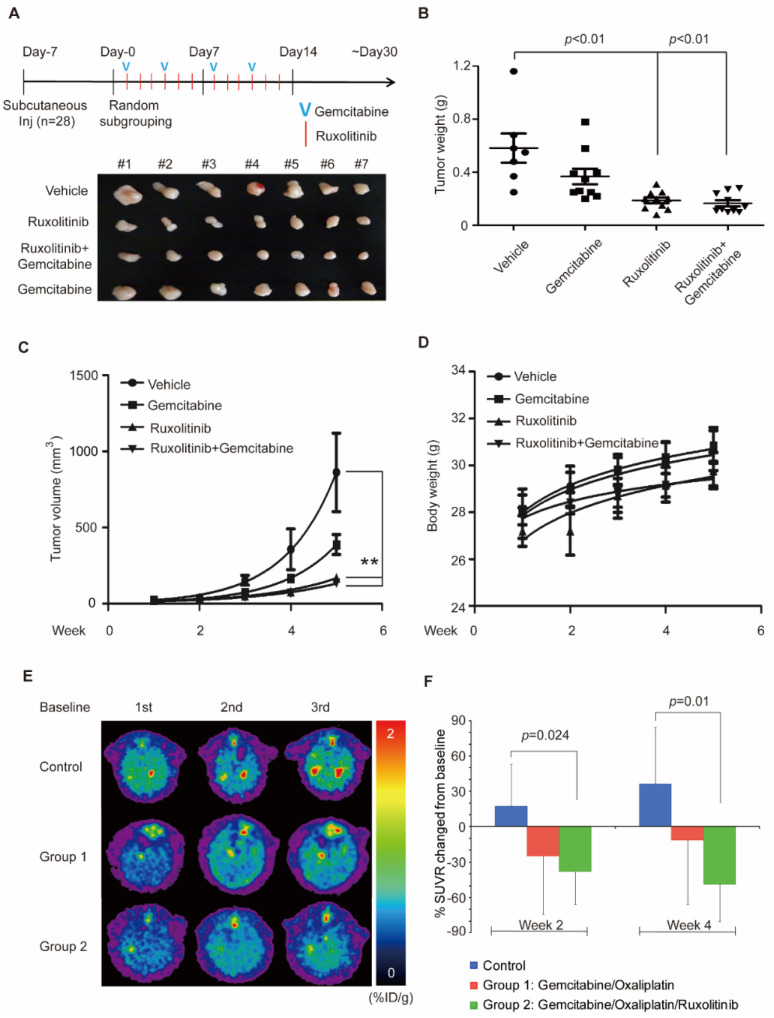
Significant inhibitory effect of ruxolitinib in vivo. (**A**) Flow chart of the xenograft animal model treated with or without gemcitabine and ruxolitinib. Representative photographs of tumor size (*n* = 7 per treatment group). Overview of (**B**) tumor weight, (**C**) tumor volume, and (**D**) body weight of mice in the sham, gemcitabine alone, ruxolitinib alone, and gemcitabine plus ruxolitinib groups. ** *p* < 0.01 compared with the vehicle cells by the paired *t* test. (**E**) Animal PET revealed a significant inhibitory effect of combined gemcitabine/oxaliplatin and ruxolitinib (a JAK-2 inhibitor) after 2- and even 4-week treatment, which was demonstrated by a significantly reduced SUV level. (**F**) The quantitative SUV levels changed from the baseline of the animal PET assay.

To determine the therapeutic efficacy of ruxolitinib alone or in combination with gemcitabine in cholangiocarcinoma, we used this animal model (Figure 4A). After randomly subgrouping the mice, decreases in tumor size (Figure 4B) and tumor volume (Figure 4C) were found in the ruxolitinib alone group and the combination group. No changes in body weight were noted for any group, suggesting that the combination therapy is not toxic for mice. Notably, we found a significantly lower SUV level (as analyzed using PET) in the group receiving ruxolitinib with gemcitabine + oxaliplatin compared with the group receiving only gemcitabine + oxaliplatin (Figure 4E,F).

### 3.5. Correlation of STAT1, STAT3, and ALDH1A3 in Patients with Cholangiocarcinoma

**Figure 5:**
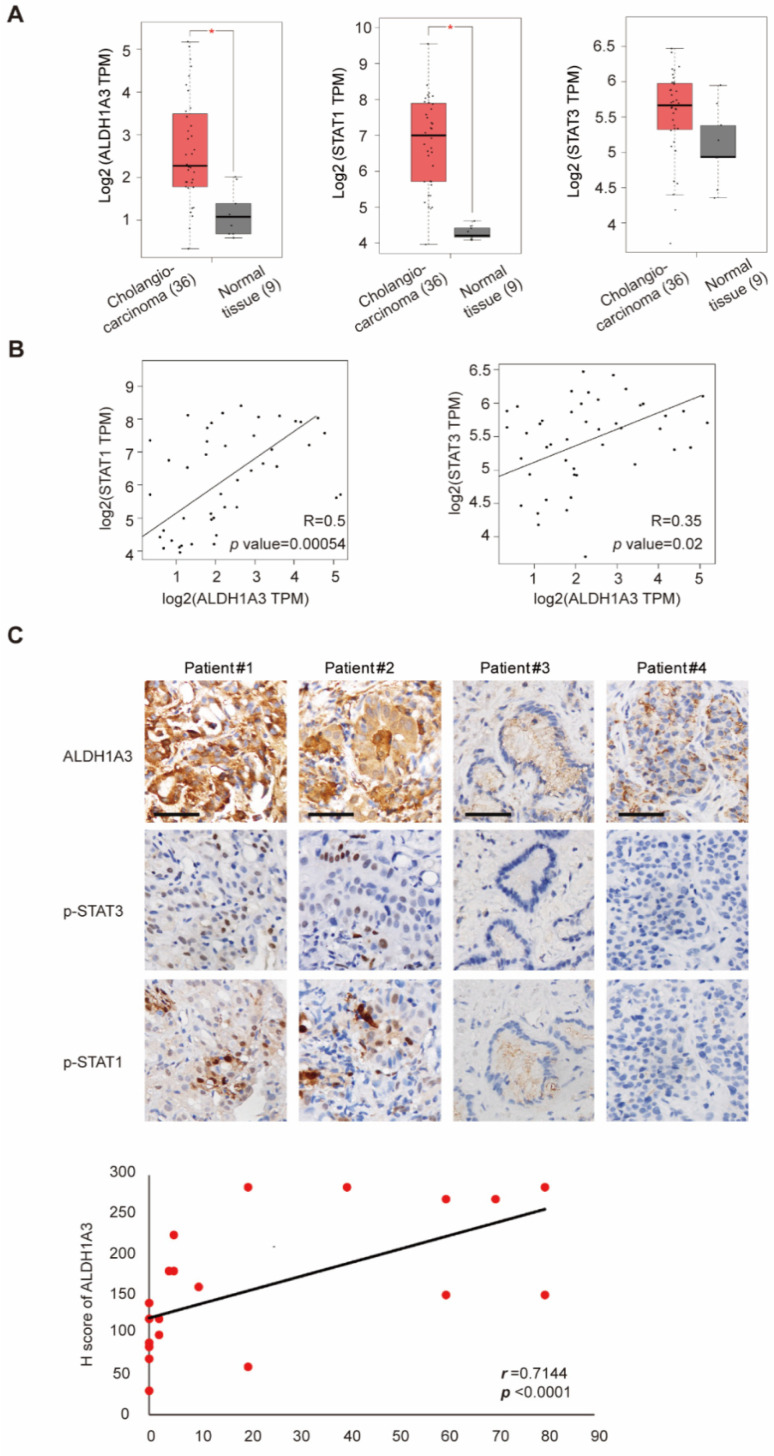
Expression of STAT1, STAT3, and ALDH1A3 in patients with cholangiocarcinoma. (**A**) Relative mRNA expressions of ALDH1A3, STAT1, and STAT3 from TCGA databank in cholangiocarcinoma (*n* = 36) and control samples (*n* = 9). * *p* < 0.05 by Mann–Whitney U test. (**B**) Spearman’s rank correlation between the mRNA levels of ALDH1A3, STAT, and STAT3. The correlation coefficients *r* and *P* values were obtained using GEPIA and are shown in each panel. (**C**) Upper: Representative imaged of immunohistochemical nuclear staining of ALDH1A3, pSTAT1 (Y701-phosphorylated STAT1), and pSTAT3 (Y705-phosphorylated STAT3) in human cholangiocarcinoma specimens. Scale bar = 50 μm. Lower: Spearman’s rank correlation coefficient *r* and *P* values are shown in the panel (*n* = 24).

We confirmed the proposed mechanisms in samples from patient with cholangiocarcinoma by using The Cancer Genome Atlas (TCGA) databank. ALDH1A3 and STAT1 mRNA levels were significantly upregulated in tumor samples (Figure 5A). Significant correlations were noted between the mRNA levels of STAT1 and ALDH1A3 and those of STAT3 and ALDH1A3 levels (Figure 5B). Among cholangiocarcinoma patient specimens, specimens with higher levels of phosphorylated STAT1 and STAT3 exhibited higher ALDH1A3 expression and vice versa (Figure 5C). This finding was consistent with previous assumptions that STAT1 and STAT3 mediate ALDH1A3 expression in cholangiocarcinoma.

## 4. Discussion

These data revealed several significant findings: First, the expression level of ALDH1A3 was highly correlated with migration ability in cholangiocarcinoma cell lines (Figure 1). Second, by using L1000, we queried gene signatures from ALDH-high cholangiocarcinoma cells and identified JAK2 inhibitor as a potential candidate (Figure 2). Third, one of the commercial JAK2 inhibitors, ruxolitinib, was found to significantly inhibit cholangiocarcinoma cell migration (Figure 3). Fourth, when combined with a gemcitabine-based chemotherapy regimen, ruxolitinib displayed a significant synergetic effect on the cholangiocarcinoma cell lines. Fifth, we propose a mechanism to explain the inhibitory effects of ruxolitinib on cholangiocarcinoma cells. Finally, we demonstrated the significant synergetic antitumor effect of ruxolitinib in an in vivo cholangiocarcinoma model.

Several studies have confirmed the critical role of ALDH1A3 in increasing metastatic behavior and cancer stemness in several cancer types, including breast cancer, lung cancer, colon cancer, glioblastoma, and cholangiocarcinoma [16,21,22,23,24,25,26]. Kim et al. reported that the activation of hypoxia-inducible factor-2α enhances breast cancer stemness [22]. Another study emphasized the vital role of ALDH1 in breast cancer metastasis and drug resistance [21]. Our previous work demonstrated that ALDH-high cholangiocarcinoma cells tend to migrate faster and are more resistant to gemcitabine. ALDH1A3 is an independent poor prognostic factor in patients with intrahepatic cholangiocarcinoma who underwent hepatectomy and those who received chemotherapy [16]. We demonstrated that the ALDH1A3 level was positively correlated with cholangiocarcinoma cell migration ability.

Gemcitabine-based chemotherapy has been the mainstay of chemotherapy for advanced cholangiocarcinoma. The FDA recently approved an FGFR2 inhibitor, pemigatinib, which demonstrated a survival benefit in patients with cholangiocarcinoma with FGFR2 rearrangement or alteration [15]. Because ALDH1A3 is highly correlated with cholangiocarcinoma cell metastatic ability and stemness, a drug that inhibits or reverses the effect of ALDH1A3 may be therapeutically advantageous. The L1000 gene expression profiling platform, an innovative gene expression profiling solution for next-generation pharmaceutical discovery applications, enabled us to effectively explore the possible candidates from the LINCS database, which consists of transcriptional expression data from 15 different cultured human cells treated with bioactive small molecules and genetic perturbations. JAK2 inhibitor was the best candidate from the detailed selection.

Several studies have discussed the ALDH isoenzyme and downstream signal transduction in various cancer types [27,28,29,30,31]. To explore how ruxolitinib, a JAK2 inhibitor, reduces ALDH1A3 expression in cholangiocarcinoma cells, we used a microarray to investigate the transcription factors that fluctuate the most when applying different ruxolitinib concentrations to a cholangiocarcinoma cell line. On the basis of the results, we hypothesized that ruxolitinib inhibits ALDH1A3 via the JAK/STAT1/3 pathway and reduces cell proliferation and migration ability. Shao et al. highlighted that the essential role of ALDH1A3 in lung cancer stemness is associated with the STAT3 pathway [30]. They also reported that the JAK2 inhibitor can reduce ALDH^+^ lung cancer cells. In their review, Duan et al. concluded that JAK2 may be a potential pathway for cancer therapy through ALDH1A3 [31]. Our study results provide an excellent in vivo proof of these hypotheses.

To the best of our knowledge, this is the first study to report that a JAK2 inhibitor can downregulate ALDH1A3 and control cholangiocarcinoma cell growth and migration. In this study, we chose a listed JAK2 inhibitor, ruxolitinib, which inhibits ALDH1A3 expression in vitro and cholangiocarcinoma cell migration. Ruxolitinib also showed significant synergetic effects in suppressing cholangiocarcinoma cell function when combined with gemcitabine. In our in vivo model, tumor size was significantly reduced in the ruxolitinib + gemcitabine combination arm. In a previous study, we reported a lower response rate with gemcitabine in patients with a higher level of ALDH1A3 [11]. According to our current findings, adding ruxolitinib may reverse this negative effect from ALDH1A3 and improve the treatment outcome. Human clinical trials are warranted to confirm the clinical efficacy.

**Figure 6:**
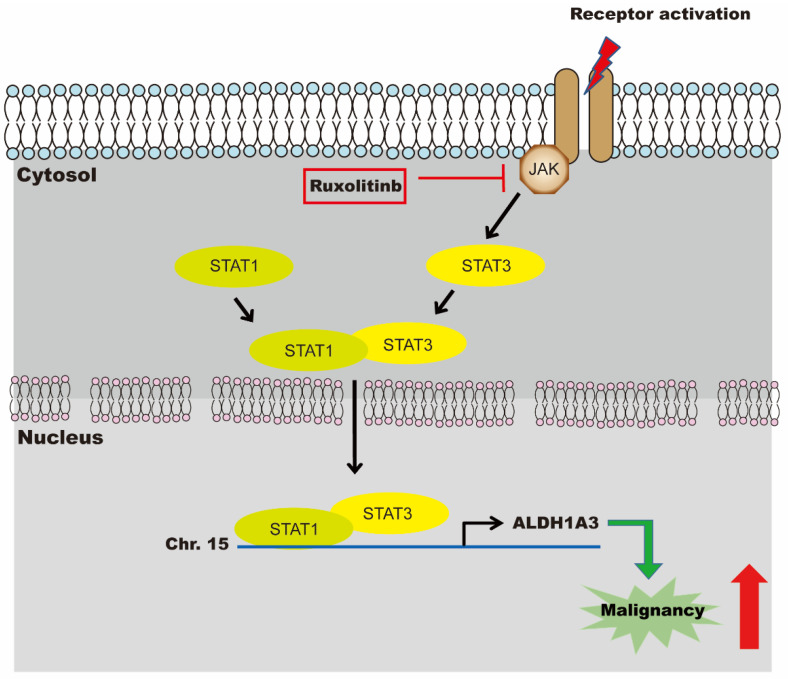
Schematic representation of the effects of ruxolitinib on malignancy of cholangiocarcinoma. A JAK2 inhibitor, ruxolitinib, can downregulate ALDH1A3 expression and inhibit cell proliferation and migration through STAT1/STAT3 in cholangiocarcinoma cell lines.

In conclusion, by using the LINCS program, we discovered that a JAK2 inhibitor, ruxolitinib, can downregulate ALDH1A3 expression and inhibit cell proliferation and migration in cholangiocarcinoma cell lines. When combined with gemcitabine, tumor size was significantly reduced in cholangiocarcinoma animal models. The inhibitory mechanism of ruxolitinib may be effected through the JAK/STAT1/3 pathway (Figure 6). Further clinical trials are warranted for efficacy in humans.

## Data Availability

The data that support the findings of this study are available from the corresponding author upon reasonable request.

## Supplementary Materials

The following are available online at https://www.mdpi.com/article/10.3390/biomedicines9080885/s1, Figure S1: The inhibitory effect of ruxolitinib in cholangiocarcinoma cell lines. Figure S2: Ruxolitinib inhibits cell migration via suppressing ALDH1A3. Figure S3: STAT3 inhibitor inhibited growth and migration in cholangiocarcinoma cell lines. Table S1: Transcription factors prediction by JAK2 inhibitor. Table S2: JAK2 canonical pathway.
