# Supplementary material for: Ruxolitinib Combined with Gemcitabine against Cholangiocarcinoma Growth via the JAK2/STAT1/3/ALDH1A3 Pathway
Source: Biomedicines. 2021 Jul 24;9(8):885. doi: 10.3390/biomedicines9080885 (PMC8389680; doi:10.3390/biomedicines9080885)
Supplement: Supplementary file 1 [file biomedicines-09-00885-s001.zip › Supplementary figure.pdf]

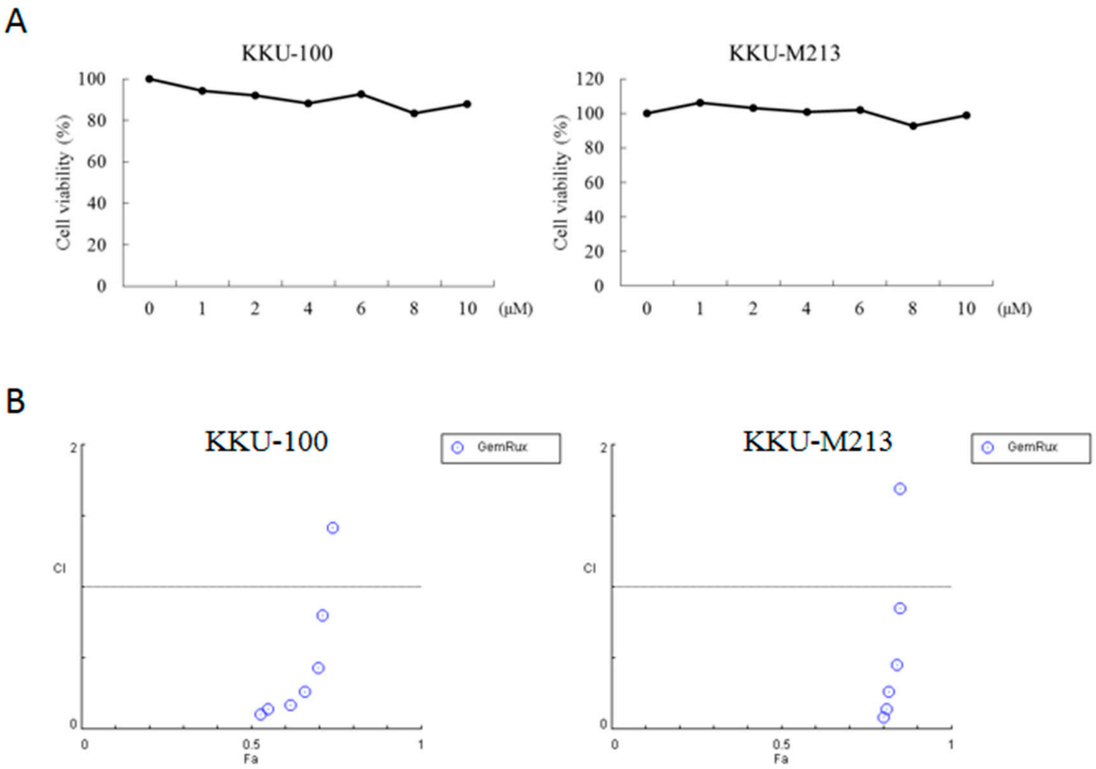

Figure S1. The inhibitory effect of ruxolitinib in cholangiocarcinoma cell lines. (A) The KKU-100 (left) and KKU-M213 (right) cells were seeded onto 96-wells plates and treated with ruxolitinib alone for 72 hours. The cell viability was measured by MTT assay and was normalized to respective untreated cells. (B) The isobologram of effect analysis for KKU-100 (left) and KKU-M213 (right). Fa: effect level; CI: combination index. CI<1: synergy effect; CI=1, additive effect; CI>1, antagonism effect.

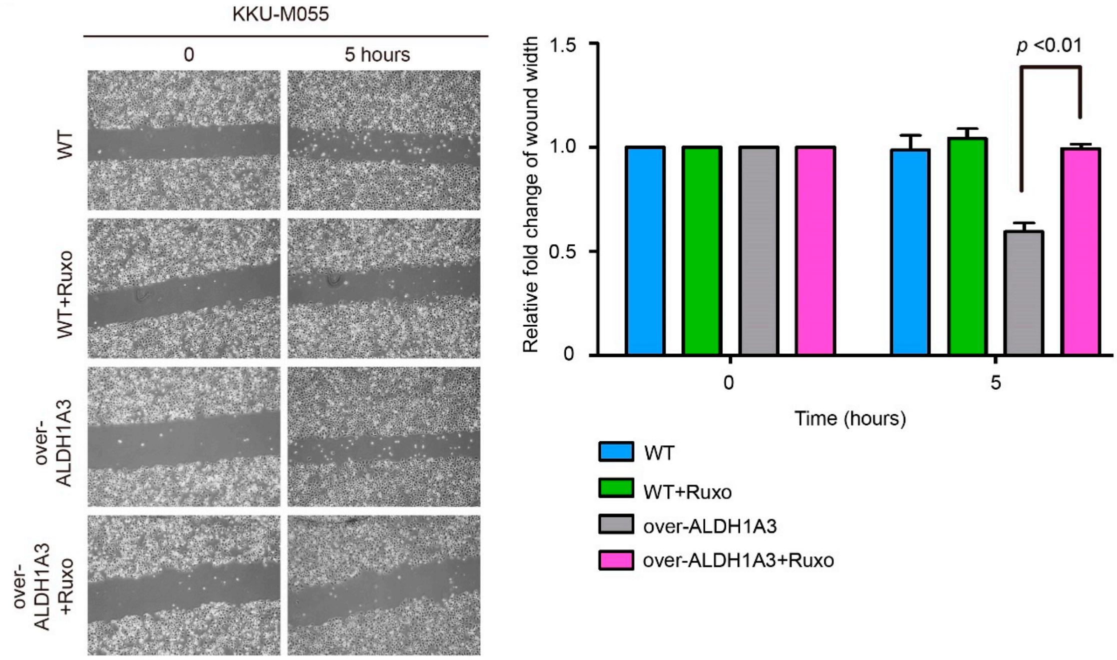

Figure S2. Ruxolitinib inhibits cell migration via suppressing ALDH1A3. Cell migration abilities were analyzed using a wound healing assay. Representative images reveal KKU-M055 cells treated with 10 µM ruxolitinib compared with the control after 0 and 5 h. The quantitative results shown in the bottom panels are the mean ± standard deviation of three independent experiments. *P < 0.05 compared with the respective vector cells by the Student's t test.

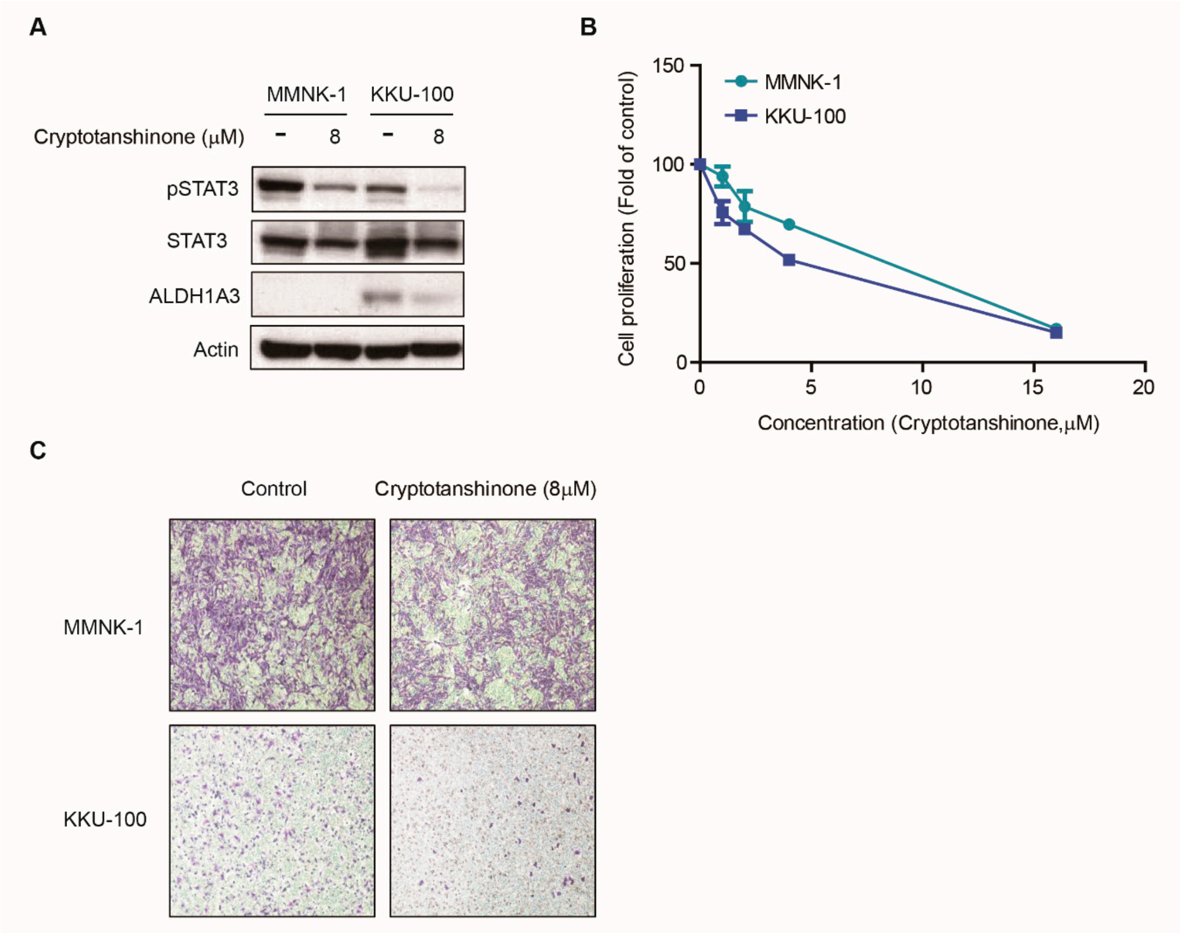

Figure S3. STAT3 inhibitor inhibited growth and migration in cholangiocarcinoma cell lines. (A) Total lysates (20 μg) prepared from MMNK-1 and KKU-100 cells treated with or without 8 μM cryptotanshinone (an STAT3 inhibitor) were subjected to Western blotting using primary antibodies against ALDH1A3, STAT3, and p-STAT3 as probes. β-actin signals were used as loading controls. (B) Effects of cryptotanshinone on cell proliferation in MMNK-1 and KKU-100 cells. At 48 h after drug treatment, cell viability was measured using an MTT assay and was normalized to the respective untreated cells. Data shown are the averages of three independent experiments. (C) Representative microphotographs of the migrated MMNK-1 and KKU-100 cells after treatment with 8 μM cryptotanshinone.
